# Identification of a Novel Human Papillomavirus by Metagenomic Analysis of Samples from Patients with Febrile Respiratory Illness

**DOI:** 10.1371/journal.pone.0058404

**Published:** 2013-03-15

**Authors:** John L. Mokili, Bas E. Dutilh, Yan Wei Lim, Bradley S. Schneider, Travis Taylor, Matthew R. Haynes, David Metzgar, Christopher A. Myers, Patrick J. Blair, Bahador Nosrat, Nathan D. Wolfe, Forest Rohwer

**Affiliations:** 1 Department of Biology, San Diego State University, San Diego, California, United States of America; 2 Global Viral Forecasting (now known as Metabiota), San Francisco, California, United States of America; 3 Centre for Molecular and Biomolecular Informatics/Nijmegen Centre for Molecular Life Sciences, Radboud University Medical Centre, Nijmegen, The Netherlands; 4 Naval Health Research Center, U.S. Navy, San Diego, California, United States of America; 5 Center for Microbial Sciences, San Diego State University, San Diego, California, United States of America; Albert Einstein College of Medicine, United States of America

## Abstract

As part of a virus discovery investigation using a metagenomic approach, a highly divergent novel *Human papillomavirus* type was identified in pooled convenience nasal/oropharyngeal swab samples collected from patients with febrile respiratory illness. Phylogenetic analysis of the whole genome and the L1 gene reveals that the new HPV identified in this study clusters with previously described gamma papillomaviruses, sharing only 61.1% (whole genome) and 63.1% (L1) sequence identity with its closest relative in the Papillomavirus episteme (PAVE) database. This new virus was named HPV_SD2 pending official classification. The complete genome of HPV-SD2 is 7,299 bp long (36.3% G/C) and contains 7 open reading frames (L2, L1, E6, E7, E1, E2 and E4) and a non-coding long control region (LCR) between L1 and E6. The metagenomic procedures, coupled with the bioinformatic methods described herein are well suited to detect small circular genomes such as those of human papillomaviruses.

## Introduction

Papillomaviruses (PV) are small, circular, double-stranded DNA viruses belonging to *Papillomaviridae*, a large family with over 180 viruses phylogenetically classified in groups, genera, and species [1], [2]. The PVs infecting human, the human PV (HPVs), encompass highly pathogenic viruses causing a wide range of diseases including cervical, vulval, penile, oropharyngeal and anal warts and cancers [3]–[6]. Over 150 genomes of HPVs have been fully sequenced, characterized and catalogued by the Papillomavirus Episteme (PAVE) database. It is likely that more unknown HPV types are yet to be discovered, and may be circulating between humans, animals and their respective environments. It is necessary to determine the full picture of the genetic diversity, the host-range and the clinical relevance of the vast majority of HPVs, which thus far remain unclear. One obstacle to discovering novel HPVs viruses has been the lack of suitable laboratory techniques and computational methods to survey environments and biological samples.

While various laboratory techniques for the detection of known HPVs are readily available, they are unpredictable for find highly divergent viruses in biological and ecological environments. Although generally viewed as the gold standard of novel virus discovery, tissue culture techniques are inefficient to propagate HPVs [2]. Therefore, the detection of HPVs primarily relies on molecular techniques such as PCR using consensus primers [7], [8], and the sequencing of a subset of the genome. The subsequent genotyping of the partial genome of HPV is generally carried out by pair-wise comparison of the sequences with the corresponding genes of known viruses. This approach can lead to analytical difficulties when non-overlapping genomic regions of different viruses are compared. Moreover, PCR using consensus primers is inadequate for highly distant and novel HPVs that can only detected by metagenomics [7]. In order to assign taxonomical classification, the PV Working Group of the International Committee on Taxonomy of Viruses recommends that researchers analyze at least the complete gene encoding the major capsid protein, L1 [1].

With the advent of metagenomics using high-throughput sequencing technology [9]–[12], it is possible to generate the full genome of phylogenetically HPVs at relatively low cost and unprecedented speed, regardless of genetic divergence from known viruses. Metagenomics is a culture-independent technique and does not depend on *a priori* knowledge of the genetic information of the organism to be detected [9]–[12].

As part of a virus discovery consortium between San Diego State University, Global Viral Forecasting and the Naval Health Research Center, we applied the metagenomic approach to pooled convenience samples obtained from patients with respiratory illness, and identified a novel gamma papillomavirus that is highly divergent compared to previously known HPVs.

## Results and Discussion

### Overall Taxonomic Classification of Metagenomic Reads

Sequencing reads generated by high-throughput sequencing were assembled with a threshold of 95% identity and at least 45 nt overlap and subjected to taxonomical classification using BLASTn against the Genbank database employing a stringent cut-off E-value of ≤10^−5^.

**Figure 1:**
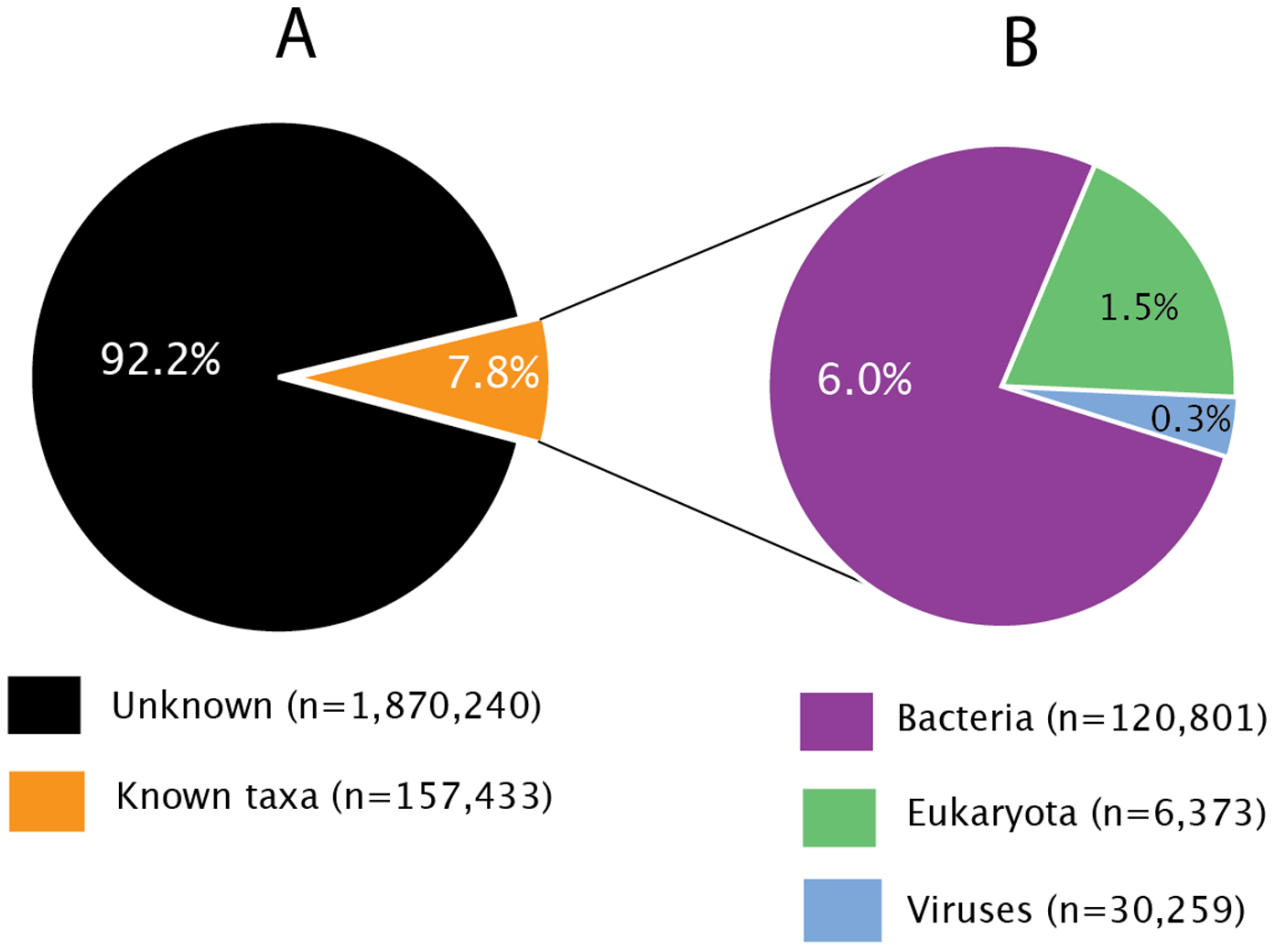
Taxonomical assignment of sequence reads convenient respiratory waste samples. A. Overall proportions of sequences with homolog in Genbank (the known, 7.8%) compared to sequences with no homolog in Genbank (Unknown, 92.2%). Unknown/Divergent indicates the proportion of highly divergent and/or novel sequences with no homology to NCBI. B. Proportion of sequences classified as eukaryotes, bacteria and viruses. Sequences were classified using BLASTn search against all non-redundant nucleotide sequences in the NCBI nt database with an E-value cutoff of 10^−5^.

The proportion of sequence reads with significant hits to viruses, bacteria and eukaryotes are summarized in Figure 1 (details provided as Document S1). Overall, of the 2,027,673 reads generated in this study, 120,801 (6%) were classified as bacterial, 30,259 (1.5%) mapped to known viruses and 6,373 (0.3%) were classified as originating from eukaryotic cells. Details of viruses identified in this study by BLASTn analysis are presented in Table 1, showing that both DNA and RNA viruses were identified in nasopharyngeal samples. These included human enterovirus, Torque teno virus, human rhinovirus, SEN virus and HPV. The HPV identified by BLAST consists of 5 contigs with significant hit to HPV_49. We combined these 5 contigs into a scaffold of length 6,356 bp, and will further refer to this scaffold as HPV_SD1 in the further analyses (below). The remaining 1,870,240 reads (92%) could not be classified as belonging to previously defined taxa. Such sequences are referred to as ‘unknowns’ throughout this paper.

**Table 1:** Summary of known viruses detected in nasopharyngeal samples.

Viral species	DNA metagenome	RNA metagenome	Total Read counts	Percentage total read
	17,313	12,946	30,259	100%
Human enterovirus C	0	9,926	9,926	36.32
Torque teno virus	8,090	0	8,090	29.61
Torque teno mini virus	3,722	0	3,722	13.62
Human rhinovirus A	0	1,531	1,531	5.60
SEN virus	1,424	0	1,424	5.21
Human rhinovirus C	0	589	589	2.16
Human papillomavirus	571	0	571	2.09
Lactobacillus phage mv4	429	0	429	1.57
Small anellovirus	254	0	254	0.93
Human rhinovirus B	245	0	245	0.90
Human rhinovirus sp.	202	0	202	0.74
Cyclovirus Chimp11	168	0	168	0.61
Human papillomavirus 49	76	0	76	0.28
TTV-like mini virus	67	0	67	0.25
Torque teno mini virus 5	32	0	32	0.12
Streptococcus phage 5093	5	0	5	0.01

BLASTn results. The new HPV-SD2 was not detected by the BLASTn analysis using a cut off E value of 10^−5^.

### Deciphering the Unknowns

Further analyses were performed to determine the origin of the unknown sequences. First, we developed a script (circular_from_ACE.pl, Document S2) to determine whether the contigs were circular. Second, any circular genomes identified were subjected to PlasMapper analysis [13] to determine if they were bacteria or plasmids. Third, all unknown sequences were tested with tBLASTx using a very relaxed E value cutoff. Finally, sequences with hits to known viruses with relaxed E value were confirmed by phylogenetic analysis.

**Figure 2:**
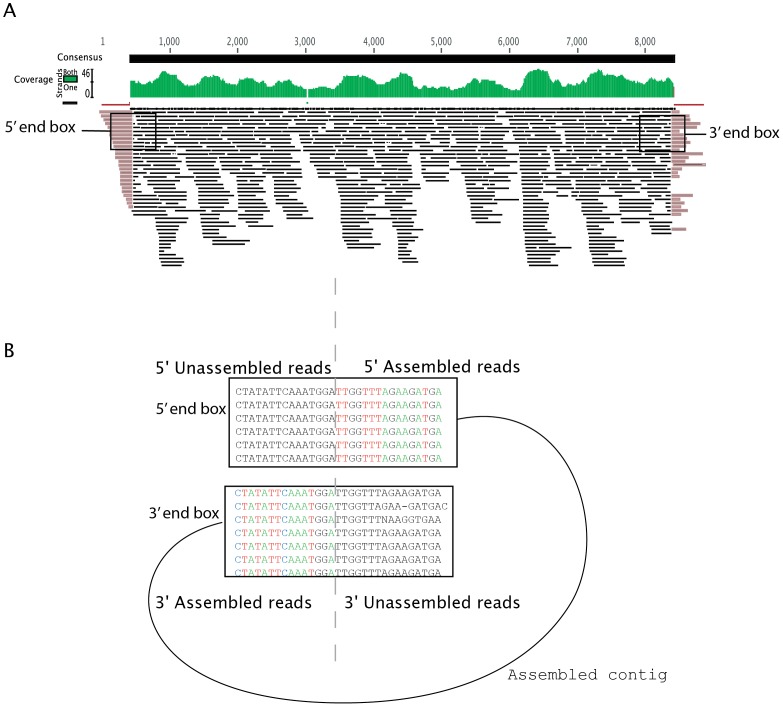
Coverage plot of the circular structure of HPV-SD2. A. Screenshot of the assembly of sequences using the *De Novo Assembler* set for 98% similarity and 45 bp overlap. The coverage plot is shown in green. Overhanging sequences at the 5′ and 3′ ends are partially assembled. B. Box at 5′ end and box at 3′ end are highlighted to show the similarity between unassembled overhang reads at 5′ and 3′ ends and corresponding ends of the consensus sequence.

The circular_from_ACE.pl script used the ACE files generated by the *De Novo* Assembler and determined whether exact copies of overhanging sequences at both 5′ and 3′ ends of the assembly were found within the contigs (Figure 2.A). Figure 2B depicts the striking similarity between overhanging fragments at the 5′ and the 3′ ends, and sequences in their respective opposite ends of the contig. Of 2,075 contigs analyzed, 193 (9.3%) met the criteria suggestive of having a circular DNA structure.

Many viral genomes including HPVs and all bacterial plasmids are characteristically circular. However, plasmids have characteristic features, including the origin of replication, which can distinguish them from viruses. The feature is seldom found among viruses, with rare exceptions such as Epstein-Barr virus, which uses an origin of replication to remain in latency in human cells [14]–[16]. Thus we investigated whether the circular genomic elements were plasmids or viruses by screening the apparently circular contigs for plasmid-specific motifs, including the origin of replication, using PlasMapper [13]. We found no evidence for these signals. The method for identification of circular genomes described here permitted us to characterize 9.3% of the unknown sequences including the putative novel papillomavirus identified in this study. Additional studies are needed to fully characterize these putative novel viral genomic structures.

All contigs were further analyzed with tBLASTx against NCBI sequences to assign taxonomy. This analysis revealed that a contig constructed with 599 sequencing reads had a significant similarity to HPV sequences. The contig was named HPV_SD2.

### Genomic Organization of and Phylogenetic Analysis of HPV_SD2

**Figure 3:**
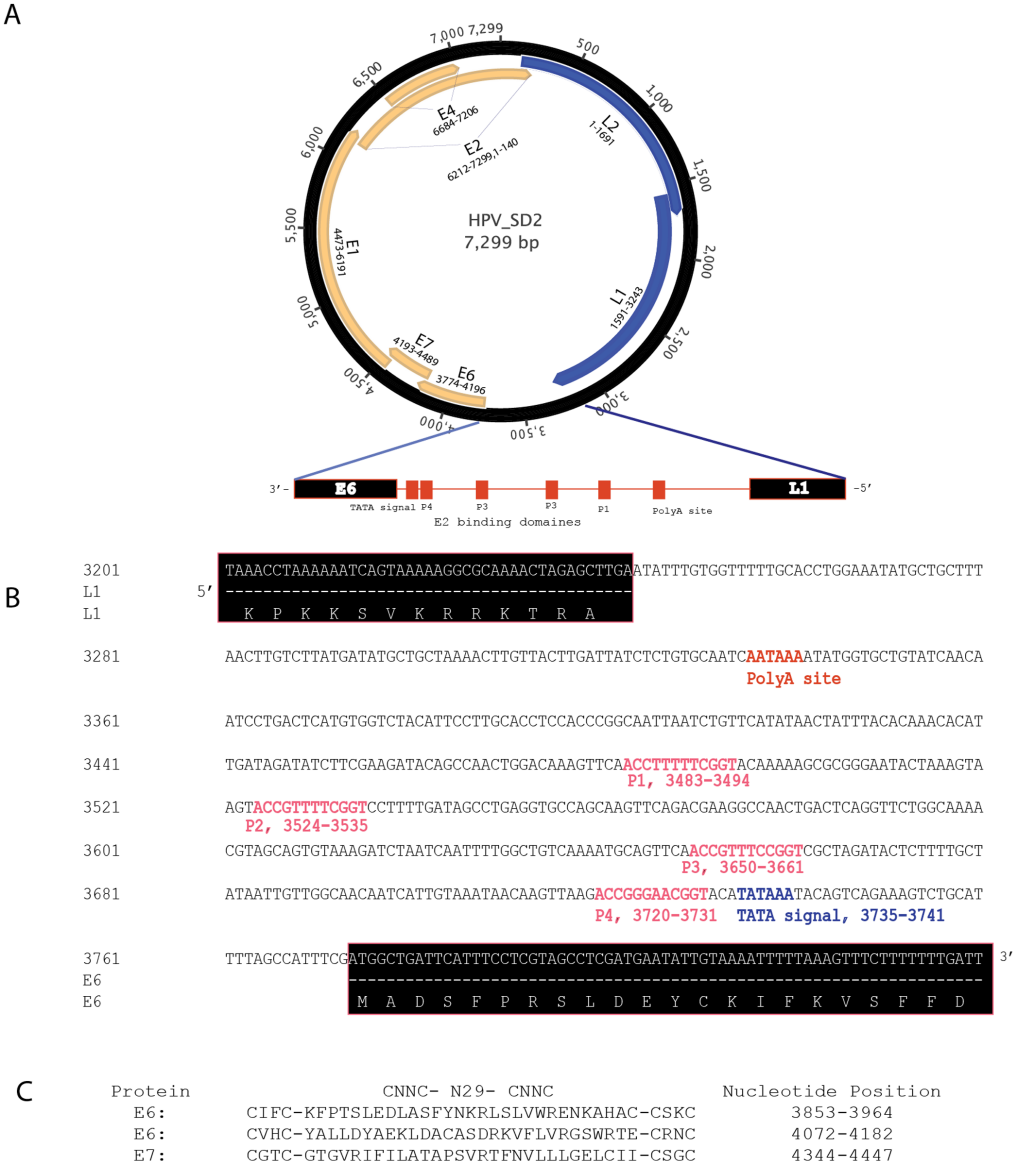
Genomic organization of the HPV_SD2 virus. Open reading frames (L2, L1, E6, E7, E1, E2, E4) and a 530 bp non-coding long control region (LCR) are shown. B. Details of the LCR region showing the TATA box (TATAAA, positions 3735–3740), a polyadenylated site (AATAAA, positions 3336–3341), 3 palindrome sites (ACCG-N_4_-CGGT; positions 3483–3494, 3650–3661, 3720–3731) and 1 degenerate palindrome (ACC-N_6_-GGT, positions 3524–3535). C. Metal-binding domains in deduced E6 and E7 proteins.

The complete genome of HPV_SD2 (Figure 3) is 7,299 bp long with a GC content of 36.3%. The genomic organization of HPV_SD2 includes 7 open reading frames (L2, L1, E6, E7, E1, E2 and E4) characteristic of other previously described HPVs. In addition, there was a 530 bp non-coding long control region (LCR), also known as an upstream regulatory region (URR) located between the L1 and E6 genes, at position 3,244 through 3,773 of the genome (Figure 3B). This LCR contains a TATA box (TATAAA, nucleotide positions 3,735–3,740), a polyadenylated site (AATAAA, nucleotide positions 3,336–3,341), 3 palindrome sites (ACCG-N_4_-CGGT; nucleotide positions 3,483–3,494; 3,650–3,661 and 3,720–3,731) and 1 degenerate palindrome (ACC-N_6_-GGT, positions 3,524–3,535). The palindrome motif ACCG-N_4_-CGGT is known to play a crucial role in binding of the E2 protein [17], [18], which mediates viral transcription in *trans* [19]. The E6 protein contained 2 zinc-binding domains (CxxC-N_29_-CxxC) at positions 3,853–3,964 and 4,072–4,182.

The E7 protein is considered as an oncogene of high-risk HPVs because it shares structural and functional similarities with known DNA viruses such as SV40 and adenovirus [17], [20]. A sequence coding for a metal-binding domain, including two Cys-X-X-Cys motifs (CGTC and CSGC) separated by 29 amino acids, was found within the deduced E7 gene at positions 4,344 through 4,447.

**Figure 4:**
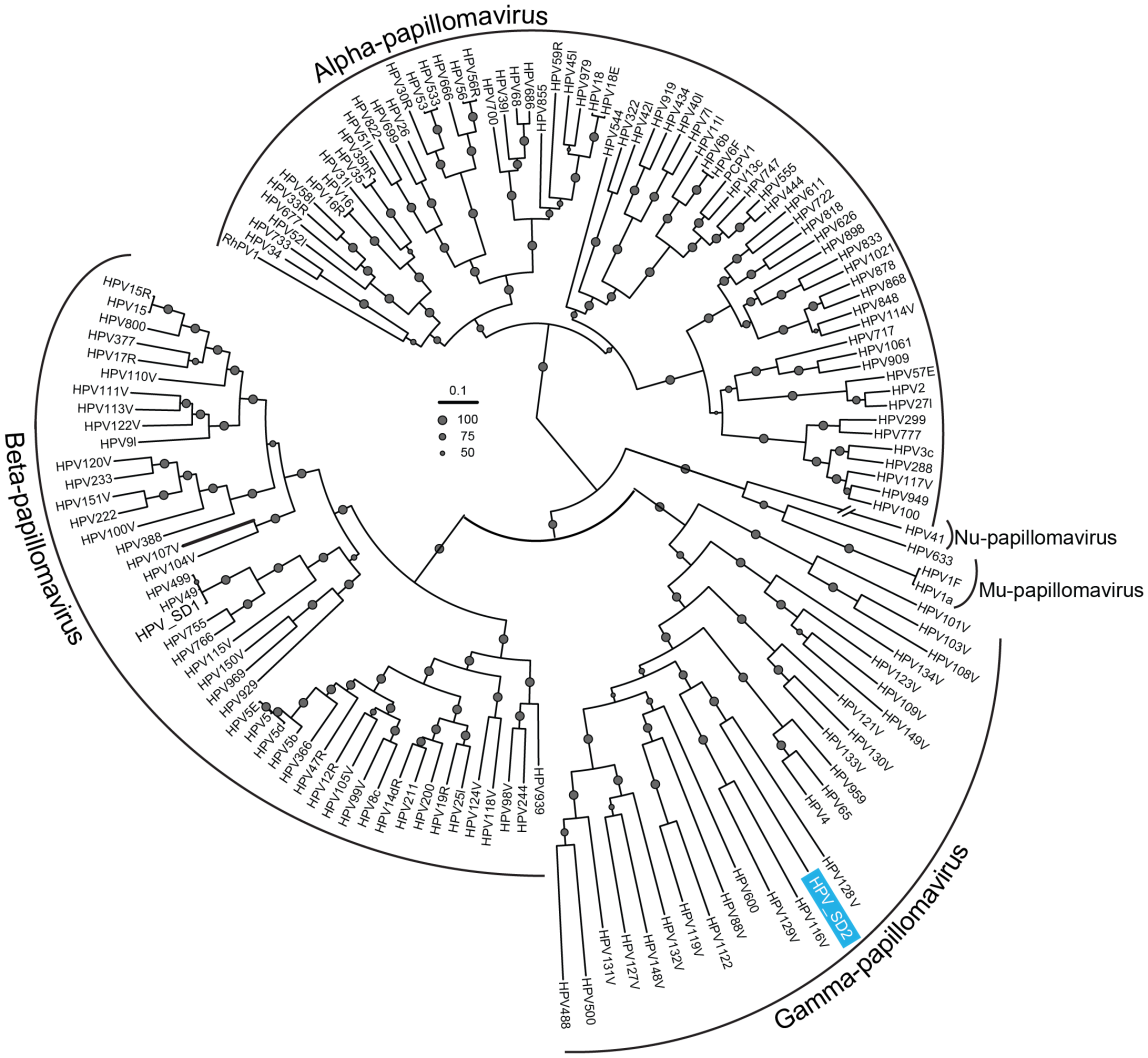
Maximum likelihood tree showing the clustering of HPV_SD2 with previously documented full-length genomes of human papillomaviruses. The tree was visualized with the Interactive Tree of Life (iTOL) [32]. Bootstrap values with at least 50%, 75% or 100% (100 re-samplings) are indicated. Note, another HPV_SD1 from this study was added in this analysis clustered with reference HPV_49.

The HPV_SD2 contig from this study were aligned with 155 complete HPV genomes in the PAVE database. The alignment was carried out using ClustalW 2.1 (default parameters) and all ambiguities and gaps were removed with GBlocks 0.91 b [21]. We also included another genome from this study, HPV_SD1, identified by BLAST analysis. The phylogenetic analysis of both the complete genome and the L1 gene showed that while HPV_SD1 was closely related to HPV_49, HPV_SD2 was a distant relative of gamma papillomaviruses. Indeed, HPV_SD2 clustered strongly with the Gamma-human papillomavirus genus, with a bootstrap value of 100% (Figure 4). A pair-wise distance analysis performed with all 155 previously described complete genome sequences and the L1 gene of papillomavirus showed that the identity between HPV_SD2 and the closest taxon (HPV128) was only 61.1% and 63.1%, respectively (Document S3). The average pair-wise identity between HPV_SD2 and all previously described complete HPV genomes depicted in Figure 4 was 49.4% (standard deviation 4.2%, Document S3).

### Verification of the Genome Structure of HPV_SD2 by PCR and Sanger Sequencing

**Figure 5:**
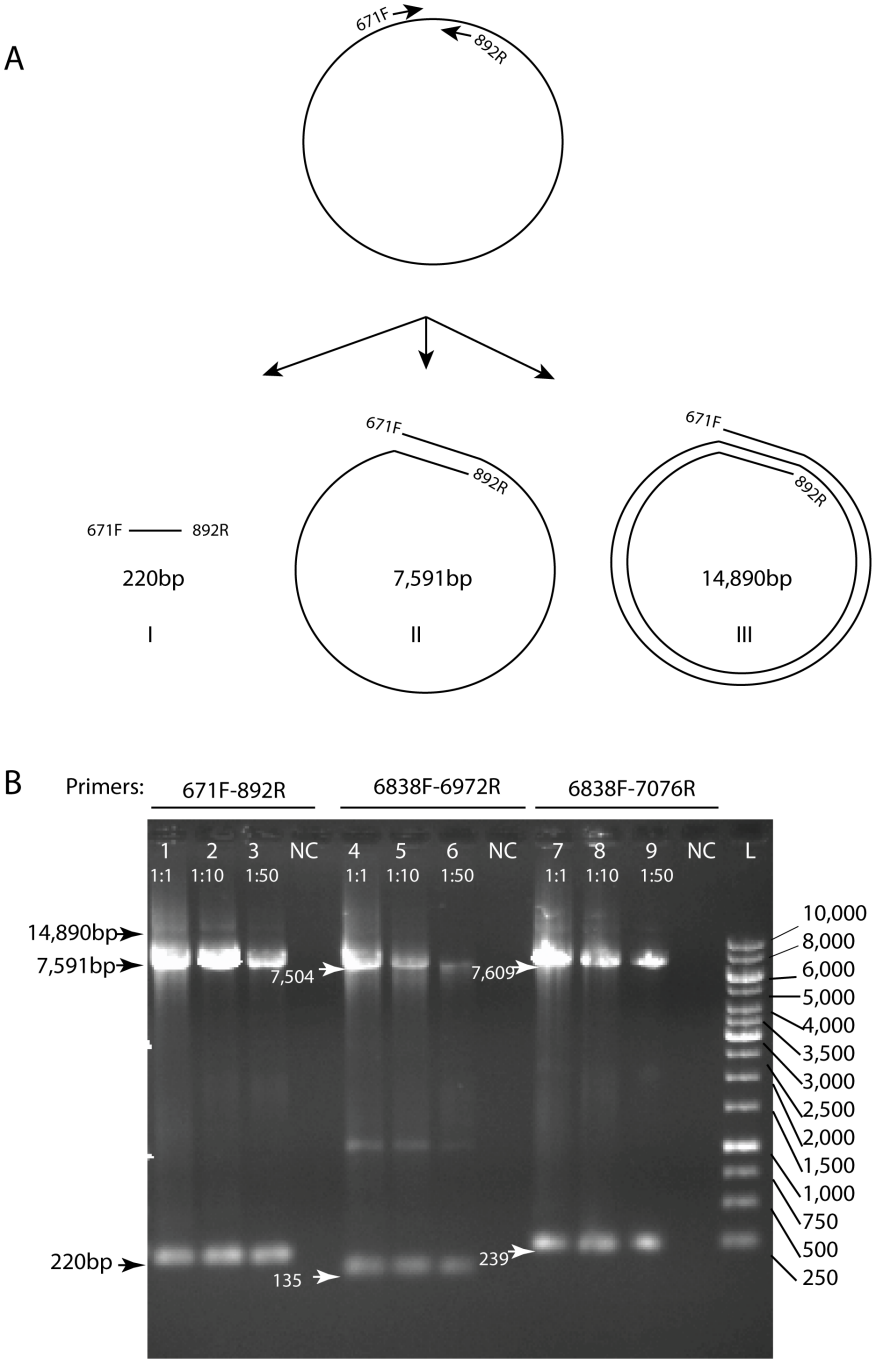
Confirmatory PCRs to verify HPV_SD2 is a circular double-stranded genome. A. Graphical representation of the binding sites of primers 671F-892R on the putative circular structure of HPV_SD2, and the predicted PCR product sizes (I: 222 bp, II: 7,591 bp, III: 14,890 bp). The predicted short band (I) indicates the amplification of the proximal region between primers 671F and 892R. The large band (II) indicates that *Taq* DNA polymerase would amplify the region between the primers 671F and 892R by making the full circle of the HPV genome. The large band (III) indicates the *Taq* DNA polymerase would make 2 full circle around the HPV genome. PCRs were also performed using primer sets 6838F-6972R, 6838F-7076R. B. Agarose gel (0.5%) showing the amplified HPV_SD2. Primer sets used are shown: primer sets 6838F-6972R, 6838F-7076R. For each PCR, the same sample pool was tested at different concentrations [1∶1 (lanes 1, 4, 7), 1∶10 (lanes 2, 5, 8) and 1∶50 (lanes (3, 6, 9)] using 1 µl per reaction. L: DNA ladder. Amplicons can be seen at the expected sizes.

We used PCR as an alternative detection method to determine if the HPV_SD2 contig generated by 454 sequencing could be found in the sample pool as a double stranded DNA. Three PCR amplifications (Figure 5A) using primer-pairs 671F-892R, 6838F-7076R and 6838F-6972R were designed to yield amplicons of 222, 135 and 239 base pairs, respectively. Because the *Taq* DNA polymerase was capable to amplify the templates beyond the primer sites, the primer sets were also expected to generate larger amplicons containing one or more full genome of HPV_SD2 (7,299 bp) plus a fragment of the size corresponding to the small amplicon product of the same primer pair (222, 135 and 239 bp). As depicted in Figure 5B. the PCR reactions with each primer pair generated the predicted amplicons of the projected sizes. The presence of these bands indicates that the HPV_SD2 is indeed a double stranded circular genome.

**Figure 6:**
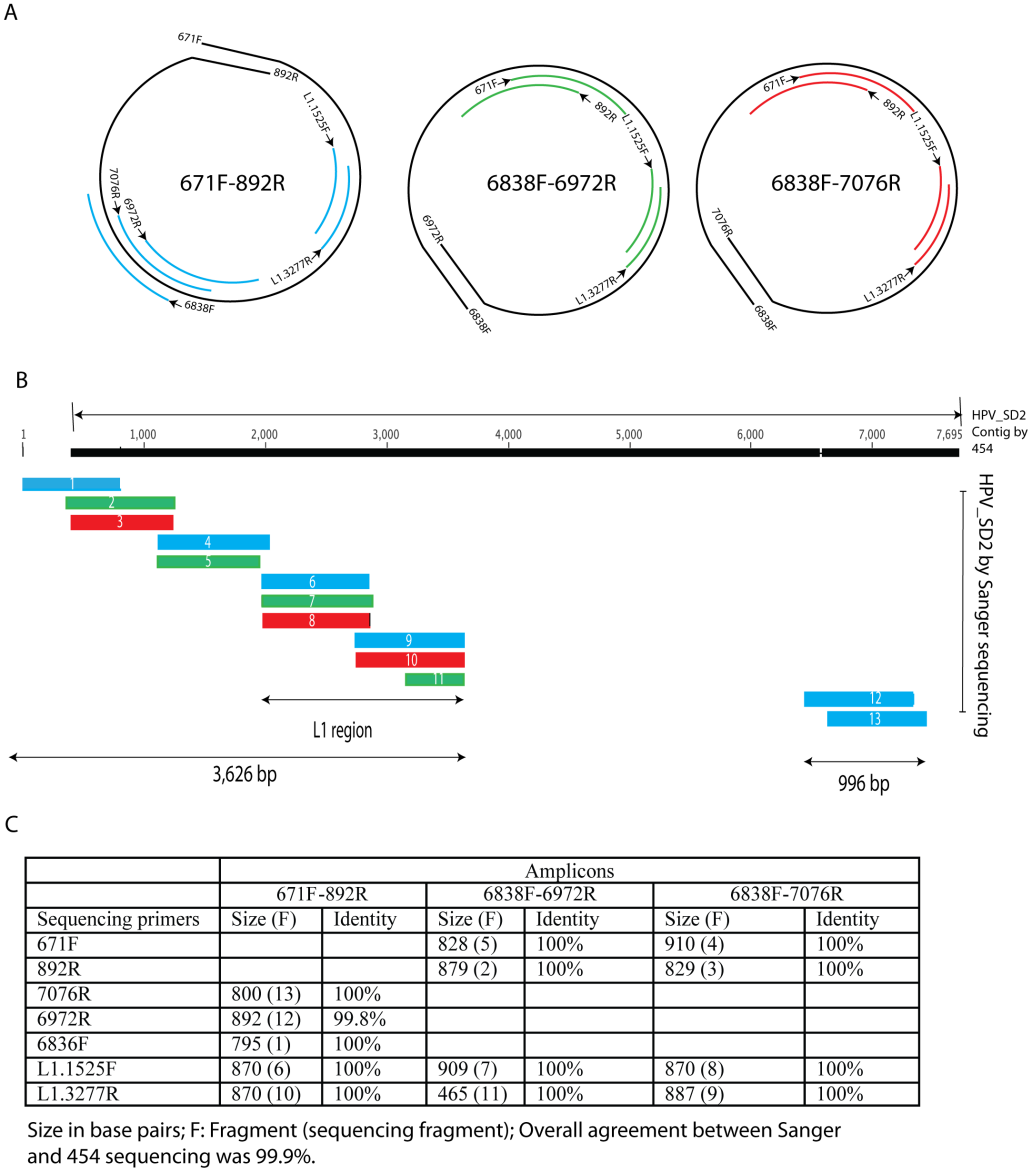
Confirmatory sequencing of full-genome amplicons generated by PCR. A. For each amplicons, the sequencing was carried out with primers other than those used for full-genome amplification. The linear sequencing template (black line), the PCR primers are shown at both ends of the amplicons. Bleu lines (amplicons 671F-892R), green lines (amplicons 6838F-6972R), red lines (amplicons 6838F-7076R) represent the sequenced regions attached to the corresponding sequencing primers. Numbers included in primer IDs represent the position relative to HPV_SD2 genome. B. Alignment of sequences generated by Sanger method along the HPV_SD2 generated by 454 sequencing. Bars in blue, green and red correspond to sequences shown in Figure 6A. The newly generated sequences covered 4,622 nucleotides representing 63.3% of the total HPV_SD2 genome. C. Comparison of the sequences generated by Sanger method and HPV_SD2 contig generated by 454 sequencing. Size of each Sanger sequence and percentage identity are shown.

To confirm the PCR products were HPV_SD2, the large amplicons containing one full genome shown in Figure 5B were purified then sequenced using the Sanger method. The resulting 13 sequences were aligned along the original contig generated by 454 sequencing (Figure 6A). The newly generated sequences covered 4,622 nucleotides including the complete L1 region of HPV_SD2 (Figure 6B). The total length of these new sequences represents 63.3% of the HPV_SD2 genome. A pair-wise comparison between the new sequences and the original contig (HPV_SD2) showed that the sequences newly generated sequences were identical to the contig generated with the 454 sequencing, except for 1 position, which had an insert. Twelve of 13 sequences were identical to the corresponding region of HPV_SD2 contig generated by 454 sequencing. Due to the presence of an insertion, one sequence generated by Sanger method had 99.8% identical position to HPV_SD2. Close look at this sequence suggested the 1 base insert was due to quality call in Sanger sequencing. Indeed, the additional insert would cause a frame-shift in the open reading frame of HPV_SD2, suggesting that the sequencing by 454 was correct. Overall, the confirmatory Sanger sequencing results were in agreement with our data by 99.9% (Figure 6C.). Therefore, the PCR amplification and sequencing confirm the discovery of a novel circular genome of human papillomavirus HPV_SD2.

### Conclusion

This study was carried out to develop a standard operating procedure for virus discovery using a metagenomic approach. To increase our sampling range, samples were tested in pools. This limited our ability to trace back any identified virus to an individual patient. Thus, we cannot link HPV_SD2 or any of the other viruses detected to clinical symptoms. Nevertheless, the metagenomic approach has been shown to be a powerful technique to detect and characterize new viruses that could have been missed by culture-dependent approaches or by sequence-dependent detection using probes. The metagenomic procedures and bioinformatic methods described in this study are suited for the detection of novel circular genomes of viruses, including unknown human papillomaviruses.

More HPV types are likely to be discovered as newer sequencing capabilities and bioinformatics procedures are being developed. Based on the L1 ORF, different papillomaviruses of the same genera and species share at least 60% and 90% pairwise sequence identity, respectively [2]. According to the definition, the observed low degree of similarity and high genetic distance to previously described HPVs (E-M.de Villiers personal communication), we propose that the HPV_SD2 genome described in this study represents a novel human papillomavirus type of the Gamma genus.

## Materials and Methods

**Figure 7:**
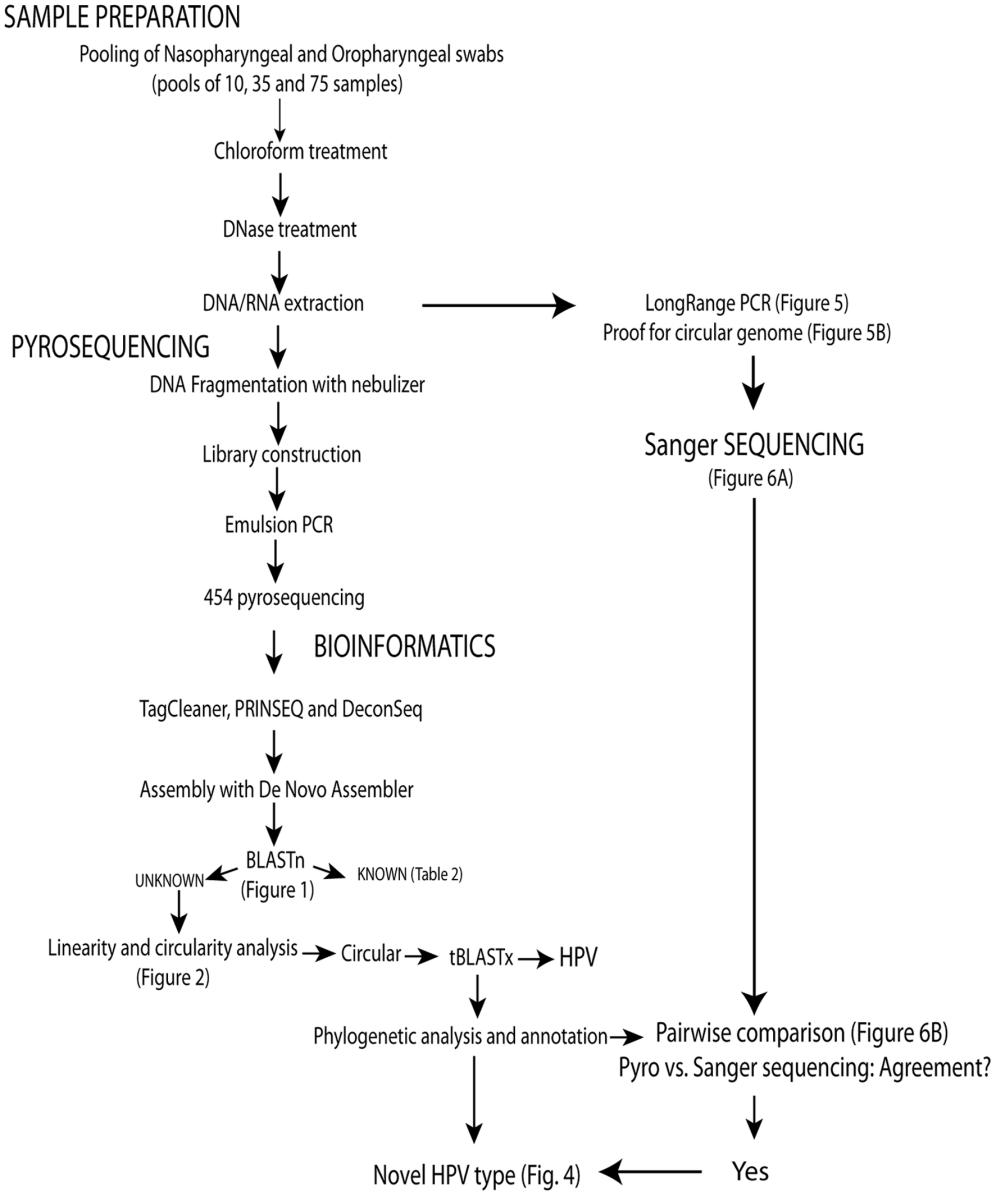
Outline of methods.

The materials and methods used in this study are outlined in Figure 7, and are detailed below.

### Ethics Statement

Convenience samples analyzed in this study were originally collected as part of a febrile respiratory illness study conducted by the US Naval Health Research Center (NHRC). The study cohort included military recruits from various training facilities throughout the United States, residents of the US/Mexico border in and near San Diego, and military dependents. That study was approved by the NHRC Institutional review board and a signed consent was obtained from all patients prior to enrollment. Samples were transferred to SDSU as part of virus discovery collaboration between NHRC, Global Viral Forecasting, Inc. and SDSU, and included patients with pneumonia, febrile respiratory illness, and healthy controls. The metagenomic study described here was approved by SDSU’s Health Service Biological Use Authorization (BUA 06-02-062R).

### Sample Preparation

Samples were collected with nasopharyngeal and oropharyngeal swabs and stored at −80°C in transport media (Remel Microtest™ M4 or Remel Microtest™ M4RT, Lenexa, KS, USA). Testing was performed in pools of 10, 35 or 75 samples using 40 µl, 100 µl and 100 µl, respectively (Table 2). Pooled samples were mixed with an equal volume of sodium magnesium solution (35.8 g/L, NaCl; 2 g/L, MgSO_4_ 7H_2_O; 12.5 mM TrisCl, pH 7.4) and filtered through 0.45 µm filters (Whatman, USA) to remove eukaryotic cells, bacteria and large debris. The filtrates containing viral particles, bacteria and cells that could not be removed by filtration, were treated with chloroform (20% of the total volume) to lyse any eukaryotic host cells and denature lipid bi-layers of enveloped viruses and bacteria. Samples were then treated with DNase I (0.7 units/µl of sample) to remove any free DNA. DNA and RNA were extracted concurrently using the QIAamp MinElute virus spin kit (Qiagen, cat. 51104, USA). The extracted materials containing both DNA and RNA was treated with RNase-free DNase I, then the RNA was reverse transcribed to cDNA using the Retroscript kit (Ambion, USA) (Libraries: LIB3RNA, LIB8RNA and LIB10RNA). Alternatively, the extraction of RNA for 2 libraries (LIB24RNA and LIB25RNA) was carried out using the Trizol-LS method according to manufacturer instructions, followed by cDNA synthesis using the TransPlex Complete Whole Transcriptome Amplification Kit (Sigma-Aldrich, Cat. WTA2, St. Louis, MO, USA). Four aliquots of each DNA and cDNA library were amplified by a phi29 DNA polymerase full-genome amplification method [22] then pooled together to minimize preferential amplification of small circular viruses. Amplified DNA and cDNA were then purified using the NucleoSpin® Tissue Kit (Clontech, Mountain View, CA 94043, USA). Epifluorescence microscopy was used to ascertain the presence of microbes and viruses before and after chloroform treatment [23].

**Table 2:** Composition of sample pools and methods for DNA and RNA extraction.

Sample Pools	Samples (n)	Library ID(Type)	Extraction method	Nucleic acid amplification method
Pool 1	10	LIB1 (DNA)	Qiamp Minelute virus spin	phi29
		LIB3 (RNA)*	Qiamp Minelute virus spin	
Pool 2	10	LIB7 (DNA)	Qiamp Minelute virus spin	phi29
		LIB8 (RNA)*	Qiamp Minelute virus spin	
Pool 3	75	LIB9 (DNA)	Qiamp Minelute virus spin	phi29
		LIB10 (RNA)*	Qiamp Minelute virus spin	
Pool 4	35	LIB24 (RNA)	Trisol-LS	WTA2
Pool 5	35	LIB25 (RNA)	Trisol-LS	WTA2

Phi29: Phi29 DNA polymerase full-genome amplification method [22].

WTA2: TransPlex Complete Whole Transcriptome Amplification Kit (Sigma-Aldrich, Cat. WTA2, St. Louis, MO, USA).

* cDNA synthesis was carried out using Retroscript kit (Ambion, USA) prior to sequencing.

Sequencing was carried out using the Roche 454 FLX titanium according to the manufacturer’s protocol (454 Life Sciences Corporation, Roche, Brandford, CT, USA). Approximately 0.5–5 µg of purified DNA was fragmented by applying a 30 psi nitrogen pressure for 1 minute. Fragmentation was not required for the cDNA amplicons, as their sizes were within the required range for optimal pyrosequencing (150–800 bp). The fragmented DNA and cDNA ligated to 2 different oligo adaptors, each specific to 3′ or 5′ ends. The DNA/cDNA-adaptor complexes were attached to beads, which were encapsulated into individual micro-reactor micelles in which the fragments were amplified by emulsion PCR (emPCR) [24]. The clonally amplified DNA and cDNA were then loaded into a picotiter plate device in the 454 FLX sequencer to generate DNA sequences [24].

### Bioinformatics and Taxonomic Classification

Prior to data analysis, all metagenomic data was pre-processed through a pipeline of software including, TagCleaner, PRINSEQ and DeconSeq [25], [26]. TagCleaner was used to remove primers added during cDNA synthesis with WTA Kit [27]. PrinSeq was used to remove low-quality sequences and artifacts. Human DNA was filtered out of each metagenome using the DeconSeq software. Sequence reads in each metagenome were analyzed either unassembled or as assembled contigs to determine the viral and microbial relative abundance and diversity of each sample pool. The taxonomic assignment of each sequence or assembled contig was carried out using BLASTn (version 2.2.18) by comparing metagenomic sequences with the GenBank non-redundant nucleotide database [28] using a threshold E-value of ≤10^−5^. In the case where several related taxa yielded an equally high scoring top hit, reads were assigned to most recent common ancestor. Scores for these ancestral clades were calculated as the sum of the scores in all daughter clades [29]. Sequences with no homology in Genbank are referred to as unknowns.

### Linearity and Circularity Analysis

Sequences from each metagenome with no significant hit to Genbank were assembled using the GS *De Novo* Assembler (454 Life Sciences Corporation, Roche, Banford, CT, USA) with requirements set at 95% identity and at least 45 bp overlaps. The assembled sequence reads and their related contigs were aligned using Geneious version 5.4 [30]. A Perl script (Document S2: circular_from_ACE.pl) was developed to determine the linearity or circularity of the unknown contigs. The analysis was performed on the ACE files generated by the *De Novo* Assembler.

### Screening for Plasmid-specific Features

The PlasMapper [13], a web server interface that automatically generates and annotates circular plasmid maps, was used to determine if circular genomes found in this study had plasmid-specific patterns including promoters, terminators and replication origins. In addition to plasmid-specific features, PlasMapper [13] and Genious Pro 5.4.3 [30] were used to identify open reading frames in circular contigs.

### Human Papillomavirus Phylogeny

Taxonomical classification was performed initially with BLASTn and tBLASTx, and contigs with significant hits to human papillomavirus were aligned with complete genomes of human papilloma virus (HPV) reference sequences obtained from the Papillomavirus Episteme (PAVE) database. HPV_SD1 and HPV_SD2 from this study were aligned with PAVE sequences using ClustalW 2.1 (default parameters) and all ambiguities and gaps were removed with GBlocks 0.91 b [21]. A phylogenetic tree was constructed using PhyML 3.0.1 [31] with the following parameters: NNI tree topology search, HKY85 model of nucleotide substitution, discrete gamma model with 4 categories, estimated proportion of invariant sites and transition/transversion ratio. Bootstrap analysis using 100 resampling iterations was performed to validate the phylogenetic assignment of previously described HPV full-length genomes and to provide quality assurance of the classification of sequences. The tree was visualized with the Interactive Tree of Life (iTOL) [32].

### Confirmation by Complete Genome Amplification and Sanger Sequencing

Three polymerase chain reaction assays were performed to verify if the sequence reads assembled by the metagenomic approach derived from a complete circular double-stranded DNA in the swab sample. We designed 5 PCR primers 671F: 5′-ACCA GGCC CAAC TCCC CCAA A-3′; 892R: 5′-GACG GTCC CGCC TTTT CTTG A-3′; 7076R: 5′-CCGT TTGG CACT GGGG GAGG-3′; 6838F: 5′-CCGC CTGC GACT CCGA AGAA-3′ and 6972R: 5′-GCGG CGAT GCGG TGGT AGTT-3′, each identical to HPV_SD2 at positions 671–691, 872–892, 7057–7076, 6838–6857, 6953–7072. Three PCR reactions were designed to amplify the HPV_SD2 using primer sets 671F-892R, 6838F-7076R and 6838F-6972R (Figure 5A). The PCR amplification was performed using the Thermo Scientific Extensor Hi-Fidelity PCR Master Mix following manufacturer instruction (Thermo Fisher Scientific, Inc.). Given the large size of the amplicons and different melting temperatures of primers, we employed a touchdown approach that allowed us to amplify all the PCR products using the same conditions. The cycling conditions were as follows: 1) Denaturation, 94°C for 2 minutes; 2) denaturation, 94°C for 10 seconds; 3) Annealing, 58°C for 30 seconds; Touch-down, −0.5°C per cycle; 4) Elongation, 68°C for 8 minutes; 5) Repeat steps 2–4 28 times; 6) Elongation, 68°C for 7 minutes; store sample at 4°C until use. A 10 µl aliquot of the PCR reaction was run in a 0.5% agarose gel and any amplified amplicon was viewed by ultra violet trans-luminescence. The sizes of the predicted amplicon for these primer sets were 222, 135 and 239 bp, respectively. Assuming that HPV_SD2 is circular (see Figure 5A), each primer set (671F-892R, 6838F-7076R and 6838F-6972R) would also generate at least one larger fragment representing one or more complete genome of HPV_SD2 (7,299 bp) plus the corresponding small amplicon (222, 135, 239 base pairs, respectively) generated by the same primer set.

The resulting full-genome linear PCR amplicons were excised from the agarose gels and then purified using Zymoclean™ Gel DNA Recovery kit according to the manufacturer’s instructions. Purified amplicons were shipped on dry ice to Eton Bioscience (5820 Oberlin Drive, Suite 100; San Diego, CA 92121) for sequencing. The sequencing was carried out using the Sanger method with primers other than those used for the full-genome amplification (Figure 5).

### Sequence Data

The HPV_SD2 was linearized inside the E2 gene at nucleotide 1087 of this gene (i.e. 140 nt before the end of the E2 gene) and deposited to Genbank under the accession id: KC113191. The raw metagenomic pyrosequencing reads were submitted to Sequencing Read Archive (SRA) database under the accession id: SRA051429.

## Supporting Information

Document S1 **Results of the BLASTn analysis of all sequence reads against the Genbank database.**
(XLSX)

Document S2 **This is a Perl script developed to determine whether a contig was circular or non-circular.**
(PL) Click here for additional data file.

Document S3 **Identity matrix.** Pair-wise comparison between 155 HPV full-genomes and L1region in PAVE database and 2 HPVs sequences from this study. Values shown represent percentage identity.
(XLSX)

## References

[1] Bernard HU, Burk RD, Chen Z, van Doorslaer K, Hausen H, et al (2010) Classification of papillomaviruses (PVs) based on 189 PV types and proposal of taxonomic amendments. Virology 401: 70–79. 20206957 10.1016/j.virol.2010.02.002 PMC3400342

[2] de Villiers EM, Fauquet C, Broker TR, Bernard HU, zur Hausen H (2004) Classification of papillomaviruses. Virology 324: 17–27. 15183049 10.1016/j.virol.2004.03.033

[3] Ramamoorthy S, Liu YT, Luo L, Miyai K, Lu Q, et al (2010) Detection of multiple human papillomavirus genotypes in anal carcinoma. Infect Agent Cancer 5: 17. 20939896 10.1186/1750-9378-5-17 PMC2964599

[4] Bartholomew DA, Luff RD, Quigley NB, Curtis M, Olson MC (2011) Analytical performance of Cervista HPV 16/18 genotyping test for cervical cytology samples. J Clin Virol 51: 38–43. 21376660 10.1016/j.jcv.2011.01.016

[5] Chaturvedi AK, Engels EA, Pfeiffer RM, Hernandez BY, Xiao W, et al (2011) Human papillomavirus and rising oropharyngeal cancer incidence in the United States. J Clin Oncol 29: 4294–4301. 21969503 10.1200/JCO.2011.36.4596 PMC3221528

[6] Jemal A, Bray F (2011) Center MM, Ferlay J, Ward E, et al (2011) Global cancer statistics. CA Cancer J Clin 61: 69–90. 21296855 10.3322/caac.20107

[7] Tse H, Tsang AK, Tsoi HW, Leung AS, Ho CC, et al (2012) Identification of a novel bat papillomavirus by metagenomics. PLoS ONE 7: e43986. 22937142 10.1371/journal.pone.0043986 PMC3427170

[8] Forslund O, Ly H, Higgins G (2003) Improved detection of cutaneous human papillomavirus DNA by single tube nested 'hanging droplet' PCR. J Virol Methods 110: 129–136. 12798239 10.1016/s0166-0934(03)00109-5

[9] Riesenfeld CS, Schloss PD, Handelsman J (2004) Metagenomics: genomic analysis of microbial communities. Annu Rev Genet 38: 525–552. 15568985 10.1146/annurev.genet.38.072902.091216

[10] Edwards RA, Rohwer F (2005) Viral metagenomics. Nat Rev Microbiol 3: 504–510. 15886693 10.1038/nrmicro1163

[11] Delwart EL (2007) Viral metagenomics. Rev Med Virol 17: 115–131. 17295196 10.1002/rmv.532 PMC7169062

[12] Mokili JL, Rohwer F, Dutilh BE (2012) Metagenomics and future perspectives in virus discovery. Curr Opin Virology 2: 1–15. 10.1016/j.coviro.2011.12.004 PMC7102772 22440968

[13] Dong X, Stothard P, Forsythe IJ, Wishart DS (2004) PlasMapper: a web server for drawing and auto-annotating plasmid maps. Nucleic Acids Res 32: W660–664. 15215471 10.1093/nar/gkh410 PMC441548

[14] Deutsch MJ, Ott E, Papior P, Schepers A (2010) The latent origin of replication of Epstein-Barr virus directs viral genomes to active regions of the nucleus. J Virol 84: 2533–2546. 20032186 10.1128/JVI.01909-09 PMC2820910

[15] Ott E, Norio P, Ritzi M, Schildkraut C, Schepers A (2011) The dyad symmetry element of Epstein-Barr virus is a dominant but dispensable replication origin. PLoS One 6: e18609. 21603652 10.1371/journal.pone.0018609 PMC3095595

[16] Sarkari F, Sanchez-Alcaraz T, Wang S, Holowaty MN, Sheng Y, et al (2009) EBNA1-mediated recruitment of a histone H2B deubiquitylating complex to the Epstein-Barr virus latent origin of DNA replication. PLoS Pathog 5: e1000624. 19834552 10.1371/journal.ppat.1000624 PMC2757719

[17] Krubke J, Kraus J, Delius H, Chow L, Broker T, et al (1987) Genetic relationship among human papillomaviruses associated with benign and malignant tumours of patients with epidermodysplasia verruciformis. J Gen Virol 68 (Pt 12): 3091–3103. 10.1099/0022-1317-68-12-3091 2826651

[18] Androphy EJ, Lowy DR, Schiller JT (1987) Bovine papillomavirus E2 trans-activating gene product binds to specific sites in papillomavirus DNA. Nature 325: 70–73. 3025749 10.1038/325070a0

[19] Spalholz BA, Yang YC, Howley PM (1985) Transactivation of a bovine papilloma virus transcriptional regulatory element by the E2 gene product. Cell 42: 183–191. 2990724 10.1016/s0092-8674(85)80114-8

[20] Figge J, Webster T, Smith TF, Paucha E (1988) Prediction of similar transforming regions in simian virus 40 large T, adenovirus E1A, and myc oncoproteins. J Virol 62: 1814–1818. 2965766 10.1128/jvi.62.5.1814-1818.1988 PMC253237

[21] Castresana J (2000) Selection of conserved blocks from multiple alignments for their use in phylogenetic analysis. Mol Biol Evol 17: 540–552. 10742046 10.1093/oxfordjournals.molbev.a026334

[22] Dean FB, Nelson JR, Giesler TL, Lasken RS (2001) Rapid amplification of plasmid and phage DNA using Phi 29 DNA polymerase and multiply-primed rolling circle amplification. Genome Res 11: 1095–1099. 11381035 10.1101/gr.180501 PMC311129

[23] Wegley L, Mosier-Boss P, Lieberman S, Andrews J, Graff-Baker A, et al (2006) Rapid estimation of microbial numbers in water using bulk fluorescence. Environ Microbiol 8: 1775–1782. 16958758 10.1111/j.1462-2920.2006.01062.x

[24] Margulies M, Egholm M, Altman WE, Attiya S, Bader JS, et al (2005) Genome sequencing in microfabricated high-density picolitre reactors. Nature 437: 376–380. 16056220 10.1038/nature03959 PMC1464427

[25] Schmieder R, Edwards R (2011) Quality control and preprocessing of metagenomic datasets. Bioinformatics 27: 863–864. 21278185 10.1093/bioinformatics/btr026 PMC3051327

[26] Schmieder R, Lim YW, Rohwer F, Edwards R (2010) TagCleaner: Identification and removal of tag sequences from genomic and metagenomic datasets. BMC Bioinformatics 11: 341. 20573248 10.1186/1471-2105-11-341 PMC2910026

[27] Tomlins SA, Mehra R, Rhodes DR, Shah RB, Rubin MA, et al (2006) Whole transcriptome amplification for gene expression profiling and development of molecular archives. Neoplasia 8: 153–162. 16611408 10.1593/neo.05754 PMC1578511

[28] Altschul SF, Gish W, Miller W, Myers EW, Lipman DJ (1990) Basic local alignment search tool. J Mol Biol 215: 403–410. 2231712 10.1016/S0022-2836(05)80360-2

[29] Trindade-Silva AE, Rua C, Silva GG, Dutilh BE, Moreira AP, et al (2012) Taxonomic and functional microbial signatures of the endemic marine sponge Arenosclera brasiliensis. PLoS ONE 7: e39905. 22768320 10.1371/journal.pone.0039905 PMC3388064

[30] Drummond AJ, Ashton B, Buxton S, Cheung M, Cooper A, et al.. (2011) Geneious v5.4.

[31] Guindon S, Gascuel O (2003) A simple, fast, and accurate algorithm to estimate large phylogenies by maximum likelihood. Syst Biol 52: 696–704. 14530136 10.1080/10635150390235520

[32] Letunic I, Bork P (2011) Interactive Tree Of Life v2: online annotation and display of phylogenetic trees made easy. Nucleic Acids Res. 10.1093/nar/gkr201 PMC3125724 21470960

